# Cholesterol Management Gaps Among Patients With Established Coronary Artery Disease Hospitalized for Acute Coronary Events

**DOI:** 10.1016/j.jacadv.2026.103207

**Published:** 2026-09-03

**Authors:** Lisandro D. Colantonio, Zhixin Wang, Stephen L. Sigal, Emily B. Levitan, Vera Bittner

**Affiliations:** a Department of Epidemiology, University of Alabama at Birmingham, Birmingham, Alabama, USA; b Christus Mount Pleasant Heart and Vascular Institute, Mt. Pleasant, Texas, USA; c Department of Medicine, Division of Cardiovascular Disease, University of Alabama at Birmingham, Birmingham, Alabama, USA

**Keywords:** adults, coronary artery disease, hydroxymethylglutaryl-CoA reductase inhibitors, LDL, lipoproteins, medication therapy management, secondary prevention

## Abstract

**Background:**

Suboptimal cholesterol management in patients with coronary artery disease (CAD) may contribute to future cardiovascular events and increased health care utilization and costs.

**Objectives:**

The objectives of the study was to assess lipid-lowering medication use and low-density lipoprotein cholesterol (LDL-C) levels in adults with CAD hospitalized for acute coronary events.

**Methods:**

We analyzed adults ≥18 years of age with CAD hospitalized for myocardial infarction or unstable angina in 2023 to 2024 in the Get With The Guidelines-CAD registry. Evidence-based lipid-lowering medication was defined as statin therapy. A secondary composite definition was use of statins, ezetimibe, or proprotein convertase subtilisin/kexin type 9 inhibitors. LDL-C thresholds examined were <70 mg/dL (primary), <100 mg/dL, and <55 mg/dL (secondary).

**Results:**

Among 34,003 patients (mean age 68 years, 71% male, 73% White), 68.4% were using a statin at admission, whereas 71.1% were using statins, ezetimibe, or proprotein convertase subtilisin/kexin type 9 inhibitors. Among 23,558 patients with documented LDL-C at admission, 42.6% had LDL-C <70 mg/dL, including 50.2% and 25.4% of those using and not using statins, respectively (adjusted prevalence ratio [aPR] associated with statin use: 1.96; 95% CI: 1.84-2.08). Women were less likely than men to have LDL-C <70 mg/dL (aPR: 0.85; 95% CI: 0.81-0.85 and 0.79; 95% CI: 0.70-0.89 among those using and not using statins, respectively). Black patients using statins were less likely to have an LDL-C <70 mg/dL than their White counterparts (aPR: 0.70; 95% CI: 0.65-0.75). In secondary analyses, 67.7% and 25.4% of patients had an LDL-C <100 mg/dL and <55 mg/dL, respectively.

**Conclusions:**

Targeted quality improvement initiatives are needed to address gaps in ambulatory cholesterol management.

Joint multisociety blood cholesterol management guidelines led by the American College of Cardiology (ACC) and the American Heart Association (AHA) recommend maximally tolerated statin therapy for patients with coronary artery disease (CAD) to lower their low-density lipoprotein cholesterol (LDL-C) and prevent future cardiovascular events.^1^^,^^2^ Acute coronary events are associated with a high cost and health care services utilization, and a risk of death and disability.^3^^,^^4^ Therefore, CAD patients not using a statin or with high LDL-C who experience an acute coronary event represent a missed opportunity to improve cardiovascular outcomes and mitigate demands on health systems.

Over the past decades, there has been a substantial increase in the prescription of high-intensity statin therapy following discharge in patients with a CAD hospitalization.^5^^,^^6^ Despite this improvement, data from the National Health and Nutrition Examination Survey (NHANES) indicate that 67.9% of U.S. adults with CAD were using a statin in 2015 to 2020, and that 26.5% had an LDL-C <70 mg/dL.^7^ However, there is little real-world evidence on cholesterol management gaps in patients with established CAD who are hospitalized for acute coronary events, a population with direct health systems implications.

The purpose of the current study was to assess cholesterol management among patients with known CAD hospitalized for an acute coronary event using contemporary data from the Get With The Guidelines (GWTG)-CAD registry. GWTG is the largest quality improvement initiative for inpatient management of cardio and neurovascular disease in the United States.^8^ Prior studies suggest that cholesterol management differs by sex and race/ethnicity.^9^, ^10^, ^11^, ^12^, ^13^ Therefore, we further investigated sex and race/ethnicity disparities in cholesterol management in CAD patients hospitalized for an acute coronary event.

## Methods

We conducted a retrospective analysis using data from the GWTG-CAD registry. The GWTG-CAD registry includes patients hospitalized at participating hospitals with a ST-segment elevation myocardial infarction (STEMI), non-STEMI, unstable angina, or cardiac or noncardiac chest pain across all U.S. census regions. All data in the GWTG-CAD registry are systematically collected by each participating hospital using an online, interactive registry tool powered by IQVIA (Cambridge, MA). Data for the registry are abstracted from multiple sources, including emergency medical services records, emergency department documentation, admission records, inpatient medical records, and discharge documentation.

Data collection on the use of statins and other lipid-lowering medications at admission started in 2023. For the current study, we analyzed data from patients aged ≥18 years with known CAD who were hospitalized for a STEMI, non-STEMI, or unstable angina in 2023 to 2024. Known CAD was defined by a prior myocardial infarction, coronary artery bypass grafting (CABG), or percutaneous coronary intervention (PCI) in the patient’s medical history. We excluded patients whose symptoms were first identified in an acute care setting (including inpatient or emergency care at the same hospital or another) or in a chronic care facility, as these may be more likely to be receiving guideline-recommended therapy. We also excluded patients on dialysis, as lipid management in this population is guided by specific recommendations.^14^ Finally, we restricted the analysis to patients with data on lipid-lowering medication use at admission.

All institutions participating in the GWTG program are required to comply with privacy regulations, and all data enterers received appropriate training. The current analysis was approved by the Institutional Review Board of the University of Alabama at Birmingham.

### Study variables

The outcome variables for the current analysis were the use of statins and LDL-C levels at hospital arrival. Data collection on prior use of lipid-lowering medication and LDL-C at arrival is optional in the GWTG-CAD registry. Information on the intensity of prior statin therapy is not available. Some patients may not tolerate statin therapy or have a contraindication.^15^ As a secondary outcome, we examined the use of statins, ezetimibe, or proprotein convertase subtilisin/kexin type 9 inhibitors (PCSK9i).

The primary LDL-C threshold was <70 mg/dL, consistent with recommendations used to guide lipid-lowering therapy in the 2018 AHA/ACC/multisociety cholesterol guideline, which was available when data for the current analysis were collected.^1^ Secondary thresholds were LDL-C <100 mg/dL and <55 mg/dL. An LDL-C <100 mg/dL was a performance measure for CAD patients retired in 2015.^16^^,^^17^ An LDL-C <55 mg/dL is recommended in the 2026 ACC/AHA/multisociety cholesterol guideline for patients with very high risk of atherosclerotic cardiovascular disease (ASCVD) events.^2^ Although data on very high ASCVD risk are not available in the GWTG-CAD registry, most patients with CAD meet very high risk criteria.^2^

Sociodemographic variables analyzed in the current study include year of hospitalization, age at admission, sex at birth, race/ethnicity (patient-defined and analyzed as a social construct), payment source, AHA geographic region, and health-related unmet social needs. Government insurance was defined as having at least 1 of the following payment sources: Medicare, Medicaid, Veterans Affairs, Civilian Health and Medical Program of the Department of Veterans Affairs, Tricare, and Indian Health Services. Private insurance includes commercial insurance, typically tied to employer-based plans. Having unmet social needs was defined by reported gaps in any of the following health-related areas: education, employment, financial strain, food, living situation/housing, mental health, personal safety, substance use disorder, transportation barriers, and utilities.

Clinical variables analyzed include body mass index (calculated using weight and height at arrival), history of smoking in the past 12 months, and medical history (ie, history of stroke, peripheral artery disease, heart failure, cancer, prior CABG, prior PCI, diabetes, dyslipidemia, and hypertension). Discharge variables analyzed include disposition and prescription of any statin and, separately, high-intensity statin therapy.

Patient-level data in the GWTG-CAD registry have been linked to hospital data. Hospital-level data used in the current study include bed size, accreditation by The Joint Commission, heart-related services (ie, adult cardiac surgery, adult cardiology, adult diagnostic catheterization, and adult interventional cardiac catheterization), and rural referral center, sole community provider, and teaching status.

### Statistical analysis

We calculated descriptive statistics for characteristics of patients using and not using a statin at admission. We also calculated the proportion of patients using a statin at admission, overall, by sex, and by race/ethnicity. We used generalized estimating equation Poisson regression with robust variance estimators to obtain prevalence ratios (PRs) for statin use at admission by sex and race/ethnicity, accounting for clustering within hospitals.^18^ Model 1 was unadjusted. Model 2 included sex and race/ethnicity adjusted for calendar year, age, and AHA geographic region. A fully adjusted model, model 3, included sex and race/ethnicity adjusted for all sociodemographic, clinical, and hospital-level variables. Model 3 also included interaction terms between sex and racial/ethnic groups to determine whether sex disparities differ by race/ethnicity. Analyses were repeated for the secondary outcome of use of a statin, ezetimibe, or a PCSK9i. Among patients who were discharged alive to their homes, we also calculated the proportion who were prescribed any statin and, separately, high-intensity statin therapy, overall, by sex, and by race/ethnicity. We used generalized estimating equation Poisson regression as described previously to compare the prescription of statin therapy at discharge by sex and race/ethnicity.

We calculated descriptive statistics for patients with LDL-C ≥70 mg/dL and <70 mg/dL. We also calculated the proportion of patients with LDL-C <70 mg/dL overall, and by statin use at admission. We compared the proportion of patients with LDL-C <70 mg/dL by sex and by race/ethnicity, stratified by statin use. We used regression models 1 to 3 described previously to obtain PRs for an LDL-C <70 mg/dL associated with statin use, sex, and race/ethnicity.^18^ There are sex and racial/ethnic differences in the use of ezetimibe and PCSK9i, which may be confounders for LDL-C levels.^19^ Therefore, model 3 included further adjustment for ezetimibe and PCSK9i use. In sensitivity analysis, aforementioned calculations were repeated for LDL-C <100 mg/dL and <55 mg/dL.

In exploratory analyses, we identified sociodemographic, clinical, and hospital-level characteristics associated with statin use and, separately, LDL-C <70 mg/dL at arrival. We also examined lipid-lowering treatment regimens at admission, including combination therapies, among patients with LDL-C <70 and ≥ 70 mg/dL, separately. Several GWTG registry variables are optional, and missing data may reflect administrative or documentation practices rather than patient characteristics. Nested models were therefore used to evaluate the potential influence of missing data, with each model containing patients with complete data for covariates included at that level of adjustment. All analyses were conducted in the Precision Medicine Platform using SAS (SAS Institute Inc).^20^

## Results

**Figure 1:**
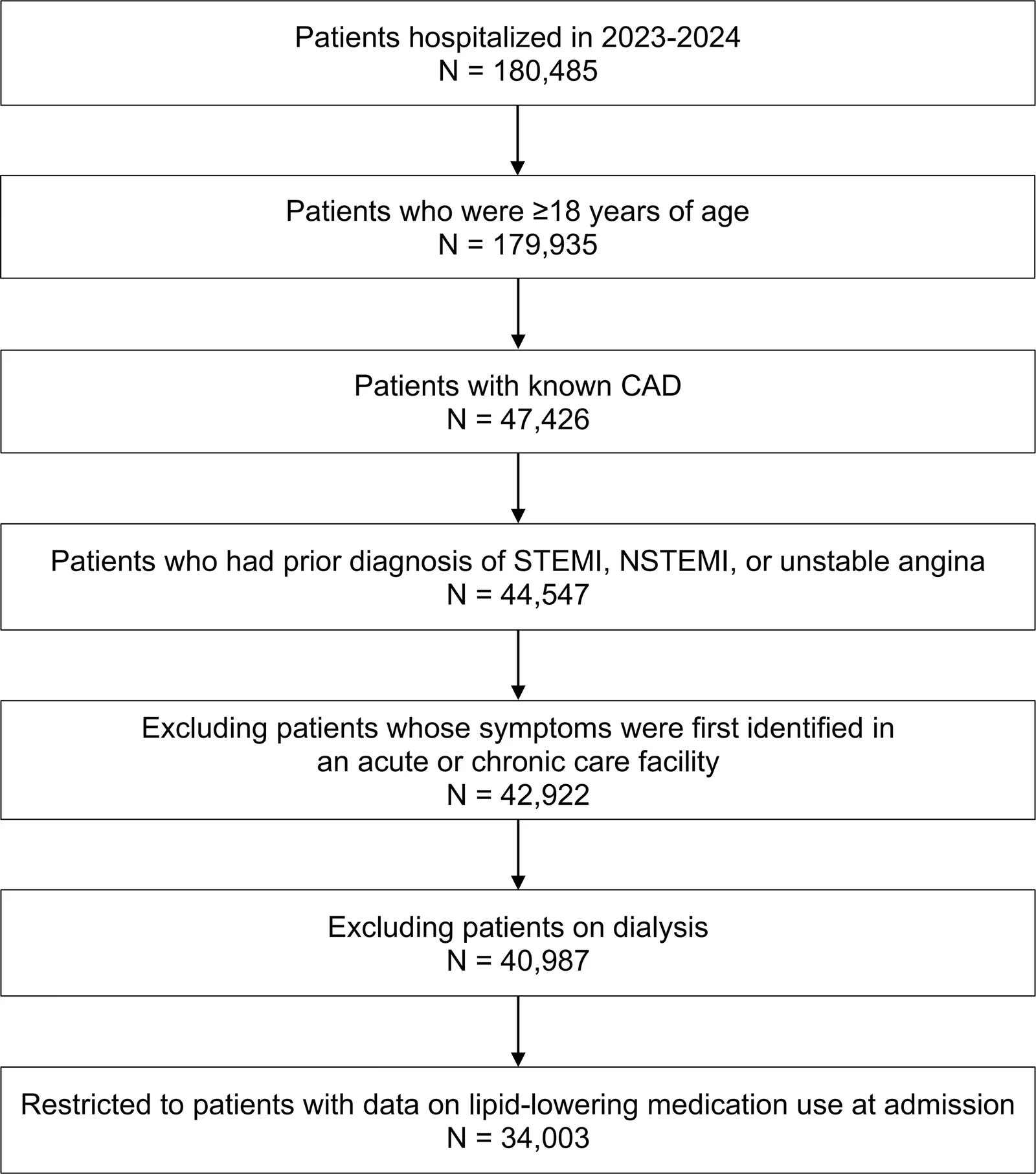
Flowchart of Get With The Guidelines-CAD Patients Included in the Current Analysis CAD = coronary artery disease; NSTEMI = non–ST-segment elevation myocardial infarction; STEMI = ST-segment elevation myocardial infarction.

The analysis included 34,003 patients from 666 hospitals (Figure 1), of whom 23,265 (68.4%) used a statin at admission. Patients using a statin were older and less likely to be from the Midwest, Western, Southeast, and Southwest regions, and to be uninsured than those not using a statin (Table 1). Patients using a statin were more likely to be from the Eastern region and have a history of peripheral artery disease, heart failure, CABG, PCI, diabetes, hypertension, and dyslipidemia.

**Table 1:** Characteristics of Patients Included in the Analysis

	Using a Statin at Admission (n = 23,265)	Not Using a Statin at Admission (n = 10,738)
Women, n (%)	6,682 (28.7)	3,153 (29.4)
Race/ethnicity, n (%)		
Non-Hispanic White	16,319 (72.2)	7,667 (74.6)
Non-Hispanic Black	3,232 (14.3)	1,276 (12.4)
Hispanic	2,023 (9.0)	906 (8.8)
Other^a^	1,018 (4.5)	431 (4.2)
Year, n (%)		
2023	11,257 (48.4)	5,189 (48.3)
2024	12,008 (51.6)	5,549 (51.7)
Age, y, mean (SD)	69.0 (11.8)	67.1 (12.6)
Age, n (%)		
18 to 44 years	549 (2.4)	422 (3.9)
45 to 59 years	4,417 (19.0)	2,502 (23.3)
60 to 69 years	6,555 (28.2)	3,140 (29.2)
70 to 79 years	7,102 (30.5)	2,819 (26.3)
≥80 years	4,642 (20.0)	1,855 (17.3)
Geographic region, n (%)		
Eastern	5,833 (25.1)	2,091 (19.5)
Midwest	3,902 (16.8)	1,837 (17.1)
Western	3,510 (15.1)	1,763 (16.4)
Southeast	4,113 (17.7)	2,209 (20.6)
Southwest	5,901 (25.4)	2,825 (26.3)
Payment source, n (%)		
Traditional Medicare	3,096 (13.6)	1,534 (14.7)
Medicare Advantage	4,904 (21.6)	1,903 (18.3)
Other government insurance^b^	5,143 (22.7)	2,220 (21.3)
Private^c^	4,583 (20.2)	2,349 (22.6)
Government and private^b^	4,000 (17.6)	1,457 (14.0)
Uninsured	798 (3.5)	796 (7.6)
Not documented	176 (0.8)	147 (1.4)
Unmet social needs, n (%)		
Yes	1,497 (6.4)	811 (7.6)
No	12,630 (54.3)	4,960 (46.2)
Not assessed	9,138 (39.3)	4,967 (46.3)
Body mass index, n (%)		
<18.5 kg/m^2^	227 (1.0)	120 (1.2)
18.5 to <25 kg/m^2^	5,036 (22.2)	2,461 (23.8)
25 to < 30 kg/m^2^	8,117 (35.8)	3,717 (35.9)
≥30 kg/m^2^	9,301 (41.0)	4,047 (39.1)
History of smoking, n (%)^d^	7,444 (32.1)	4,069 (38.1)
History of stroke, n (%)	2,845 (12.2)	1,016 (9.5)
History of PAD, n (%)	2,699 (11.6)	771 (7.2)
History of heart failure, n (%)	6,594 (28.3)	2,041 (19.0)
History of cancer, n (%)	2,746 (11.8)	954 (8.9)
Prior CABG, n (%)	6,803 (29.2)	2,442 (22.7)
Prior PCI, n (%)	17,350 (74.6)	7,255 (67.6)
Diabetes, n (%)	11,788 (50.7)	4,039 (37.6)
Dyslipidemia, n (%)^e^	19,485 (83.8)	6,836 (63.7)
Hypertension, n (%)	20,924 (89.9)	8,577 (79.9)
Using ezetimibe, n (%)	1,955 (8.4)	593 (5.5)
Using PCSK9i, n (%)	250 (1.1)	391 (3.6)
LDL-C, mg/dL, mean (SD)^f^	78.8 (42.0)	104.1 (47.6)
LDL-C, n (%)^f^		
<70 mg/dL	8,180 (50.2)	1,848 (25.4)
70 to < 100 mg/dL	4,189 (25.7)	1,743 (24.0)
≥100 mg/dL	3,925 (24.1)	3,673 (50.6)
LDL-C not documented	6,971 (30.0)	3,474 (32.4)
Hospital-level characteristics		
Bed size (categories), n (%)		
Less than 100 beds	1,621 (7.2)	1,261 (12.1)
100-199 beds	4,746 (21.0)	2,291 (21.9)
200-299 beds	5,456 (24.1)	2,390 (22.9)
300-399 beds	2,878 (12.7)	1,238 (11.9)
400-499 beds	1,940 (8.6)	882 (8.4)
500 or more beds	5,993 (26.5)	2,382 (22.8)
Accreditation by The Joint Commission, n (%)	18,797 (83.0)	8,540 (81.8)
Adult cardiac surgery, n (%)	14,459 (73.4)	6,140 (68.1)
Adult cardiology services, n (%)	18,900 (96.0)	8,442 (93.6)
Adult diagnostic catheterization, n (%)	18,614 (94.5)	8,158 (90.5)
Adult interventional cardiac catheterization, n (%)	18,589 (94.4)	8,111 (90.0)
Rural referral center, n (%)	7,923 (35.0)	3,171 (30.4)
Sole community provider, n (%)	667 (2.9)	333 (3.2)
Teaching status, n (%)^g^		
Major	3,770 (16.7)	1,422 (13.6)
Minor	14,425 (63.7)	6,354 (60.8)
Nonteaching	4,439 (19.6)	2,668 (25.5)

CABG = coronary artery bypass grafting; LDL-C = low-density lipoprotein cholesterol; PAD = peripheral artery disease; PCI = percutaneous coronary intervention; PCSK9i = proprotein convertase subtilisin/kexin type 9 inhibitor.

a The other race category includes American Indian or Alaska Native (n = 299), Asian (n = 1,225), and Native Hawaiian or Pacific Islander (n = 154).

b Government insurance includes Medicare, Medicaid, Veterans Affairs, Civilian Health and Medical Program of the Department of Veterans Affairs (CHAMPVA), Tricare, and Indian Health Services.

c Private insurance includes commercial insurance (not Medicare/Medicaid) typically tied to employer-based plans.

d In the past 12 months.

e Includes history of familial hypercholesterolemia.

f Among those whose LDL-C was documented.

g Teaching status: major teaching hospitals - those with Council of Teaching Hospitals designation (COTH); minor teaching hospitals - those approved to participate in residency and/or internship training by the Accreditation Council for Graduate Medical Education (ACGME), or the American Osteopathic Association (AOA); or those with medical school affiliation reported to the American Medical Association (AMA); nonteaching hospitals - those without COTH, ACGME, AOA or Medical School (AMA) affiliation.

Statin use by sex and race/ethnicity is shown in Table 2. After full multivariable adjustment (model 3), the PR for statin use comparing women vs men was 0.96 (95% CI: 0.94-0.99). The fully adjusted PR for statin use comparing non-Hispanic Black vs non-Hispanic White patients was 1.05 (95% CI: 1.01-1.08). No other racial/ethnic differences were statistically significant after full adjustment. There was no evidence of sex-race/ethnicity interaction (*P* = 0.41).

**Table 2:** Prevalence Ratios for Statin Use at Admission Associated With Sex and Race/Ethnicity (N = 34,003)

	Statin Use, n (%)	Prevalence Ratio (95% CI)		
		Model 1	Model 2	Model 3^a^
Overall	23,265 (68.4)	–	–	–
Sex				
Men	16,579 (68.6)	1.00 (ref)	1.00 (ref)	1.00 (ref)
Women	6,682 (67.9)	0.99 (0.97-1.01)	0.97 (0.96-0.99)	0.96 (0.94-0.99)
Race/ethnicity				
Non-Hispanic White	16,319 (68.0)	1.00 (ref)	1.00 (ref)	1.00 (ref)
Non-Hispanic Black	3,232 (71.7)	1.05 (1.02-1.09)	1.08 (1.05-1.11)	1.05 (1.01-1.08)
Hispanic	2,023 (69.1)	1.02 (0.98-1.05)	1.04 (1.00-1.08)	1.00 (0.95-1.04)
Other	1,018 (70.3)	1.03 (0.99-1.08)	1.05 (1.01-1.09)	1.01 (0.97-1.06)

Model 1 is unadjusted.

Model 2 includes sex and race/ethnicity, simultaneously, with adjustment for calendar year, age, and geographic region.

Model 3 includes sex and race/ethnicity, with adjustment for calendar year, age, geographic region, payment source, unmet social needs, body mass index, history of smoking, stroke, PAD, heart failure, and cancer, prior CABG and PCI, diabetes, dyslipidemia, hypertension, and hospital-level characteristics, including bed size, accreditation by The Joint Commission, adult cardiac surgery, adult cardiology services, adult diagnostic catheterization, adult interventional cardiac catheterization, rural referral center, sole community provider, and teaching Status. Model 3 also includes interaction terms between sex and racial/ethnic groups to determine whether sex disparities differ by race/ethnicity.

a *P* value for the interaction between sex and racial/ethnic groups: 0.41.

In the secondary analysis evaluating use of a statin, ezetimibe, or PCSK9i, 24,167 patients (71.1%) were using at least 1 of these medications. There were no statistically significant differences in the use of a statin, ezetimibe, or PCSK9i by sex or race/ethnicity (Supplemental Table 1).

Among the 25,062 patients discharged alive to their homes, 93.6% were prescribed a statin, and 85.3% were prescribed high-intensity statin therapy. Prescription of a statin and high-intensity statin therapy were each less common in women than in men (fully adjusted PR: 0.95; 95% CI: 0.94-0.97 and 0.93; 95% CI: 0.91-0.95, respectively). Prescription of a statin and high-intensity statin therapy were each more common among non-Hispanic Black (fully adjusted PR 1.02; 95% CI: 1.01-1.03 and 1.04; 95% CI: 1.02-1.05, respectively) and Hispanic patients (fully adjusted PR: 1.03; 95% CI: 1.02-1.04 and 1.05; 95% CI: 1.03-1.07, respectively) than their non-Hispanic white counterparts (Supplemental Table 2).

### LDL-C levels

Among 23,558 patients with LDL-C documented at arrival, 10,028 (42.6%) had LDL-C <70 mg/dL. Patients with LDL-C <70 mg/dL were older and less likely to be women, non-Hispanic Black, and uninsured than those with LDL-C ≥70 mg/dL (Table 3). Patients with LDL-C <70 mg/dL were also more likely to have a prior CABG or diabetes. Patients without LDL-C documented were more likely to have been hospitalized in small (<100 beds), nonteaching hospitals than those with LDL-C documented (Supplemental Table 3).

**Table 3:** Characteristics of Patients With LDL-C <70 mg/dL and ≥70 mg/dL

	LDL-C < 70 mg/dL (n = 10,028)	LDL-C ≥ 70 mg/dL (n = 13,530)
Women, n (%)	2,592 (25.9)	4,033 (29.8)
Race/ethnicity, n (%)		
Non-Hispanic White	7,444 (76.5)	9,002 (68.8)
Non-Hispanic Black	995 (10.2)	2,208 (16.9)
Hispanic	846 (8.7)	1,307 (10.0)
Other^a^	450 (4.6)	560 (4.3)
Year, n (%)		
2023	4,775 (47.6)	6,631 (49.0)
2024	5,253 (52.4)	6,899 (51.0)
Age, mean (SD)	70.8 (11.1)	66.0 (12.2)
Age, n (%)		
18 to 44 years	142 (1.4)	548 (4.1)
45 to 59 years	1,435 (14.3)	3,535 (26.1)
60 to 69 years	2,737 (27.3)	4,144 (30.6)
70 to 79 years	3,465 (34.6)	3,366 (24.9)
≥80 years	2,249 (22.4)	1,937 (14.3)
Geographic region, n (%)		
Eastern	2,489 (24.8)	3,342 (24.7)
Midwest	1,638 (16.3)	2,024 (15.0)
Western	1,632 (16.3)	1,982 (14.7)
Southeast	1,879 (18.7)	2,761 (20.4)
Southwest	2,387 (23.8)	3,410 (25.2)
Payment source, n (%)		
Traditional Medicare	1,380 (14.1)	1,625 (12.2)
Medicare Advantage	2,312 (23.6)	2,473 (18.6)
Other government insurance^b^	2,015 (20.5)	2,993 (22.6)
Private^c^	1,814 (18.5)	3,235 (24.4)
Government and private^b^	1,959 (20.0)	1,902 (14.3)
Uninsured	280 (2.9)	907 (6.8)
Not documented	48 (0.5)	135 (1.0)
Unmet social needs, n (%)		
Yes	560 (5.6)	1,129 (8.3)
No	5,888 (58.7)	7,395 (54.7)
Not assessed	3,580 (35.7)	5,006 (37.0)
Body mass index, n (%)		
<18.5 kg/m^2^	93 (0.9)	128 (1.0)
18.5 to <25 kg/m^2^	2,286 (23.2)	2,904 (21.9)
25 to < 30 kg/m^2^	3,622 (36.8)	4,704 (35.4)
≥30 kg/m^2^	3,836 (39.0)	5,541 (41.7)
History of smoking, n (%)^d^	2,724 (27.2)	5,145 (38.1)
History of stroke, n (%)	1,185 (11.8)	1,464 (10.8)
History of PAD, n (%)	1,126 (11.2)	1,236 (9.1)
History of heart failure, n (%)	2,653 (26.5)	3,059 (22.6)
History of cancer, n (%)	1,321 (13.2)	1,214 (9.0)
Prior CABG, n (%)	3,016 (30.1)	3,204 (23.7)
Prior PCI, n (%)	7,490 (74.7)	9,980 (73.8)
Diabetes, n (%)	5,130 (51.2)	5,764 (42.6)
Dyslipidemia, n (%)^e^	8,293 (82.7)	10,540 (77.9)
Hypertension, n (%)	9,004 (89.8)	11,607 (85.8)
Using a statin, n (%)	8,180 (81.6)	8,114 (60.0)
Using ezetimibe, n (%)	957 (9.5)	916 (6.8)
Using PCSK9i, n (%)	273 (2.7)	205 (1.5)
Hospital-level characteristics		
Bed size (categories), n (%)		
Less than 100 beds	345 (3.5)	498 (3.8)
100-199 beds	2,147 (22.0)	2,570 (19.5)
200-299 beds	2,238 (22.9)	3,177 (24.1)
300-399 beds	1,338 (13.7)	1,712 (13.0)
400-499 beds	910 (9.3)	1,218 (9.3)
500 or more beds	2,781 (28.5)	3,985 (30.3)
Accreditation by The Joint Commission, n (%)	8,287 (84.9)	11,243 (85.4)
Adult cardiac surgery, n (%)	6,649 (77.5)	8,975 (76.6)
Adult cardiology services, n (%)	8,458 (98.6)	11,563 (98.7)
Adult diagnostic catheterization, n (%)	8,418 (98.1)	11,515 (98.3)
Adult interventional cardiac catheterization, n (%)	8,427 (98.2)	11,503 (98.2)
Rural Referral Center, n (%)	3,513 (36.0)	4,567 (34.7)
Sole Community Provider, n (%)	214 (2.2)	261 (2.0)
Teaching Status, n (%)^f^		
Major	1,732 (17.7)	2,579 (19.6)
Minor	6,366 (65.2)	8,290 (63.0)
Nonteaching	1,661 (17.0)	2,291 (17.4)

Abbreviations as in Table 1.

a The other race category includes American Indian or Alaska Native, Asian, and Native Hawaiian or Pacific Islander.

b Government insurance includes Medicare, Medicaid, Veterans Affairs, Civilian Health and Medical Program of the Department of Veterans Affairs (CHAMPVA), Tricare, and Indian Health Services.

c Private insurance includes commercial insurance (not Medicare/Medicaid) typically tied to employer-based plans.

d In the past 12 months.

e Includes history of familial hypercholesterolemia.

f Teaching status: major teaching hospitals - those with Council of Teaching Hospitals designation (COTH); minor teaching hospitals - those approved to participate in residency and/or internship training by the Accreditation Council for Graduate Medical Education (ACGME), or the American Osteopathic Association (AOA); or those with medical school affiliation reported to the American Medical Association (AMA); nonteaching hospitals - those without COTH, ACGME, AOA or Medical School (AMA) affiliation.

Among patients not using and using a statin, 25.4% and 50.2%, respectively, had LDL-C <70 mg/dL (Table 4). After full multivariable adjustment, the PR for LDL-C <70 mg/dL associated with statin use was 1.96 (95% CI: 1.84-2.08). Women were less likely than men to have LDL-C <70 mg/dL among both those using and not using a statin (fully adjusted PR: 0.85; 95% CI 0.81-0.89 and 0.79; 95% CI 0.70-0.89, respectively). Non-Hispanic Black and Hispanic patients were less likely to have LDL-C <70 mg/dL than their non-Hispanic White counterparts among those using a statin (fully adjusted PR: 0.70; 95% CI: 0.65-0.75 and 0.91; 95% CI: 0.84-0.99, respectively). There were no statistically significant differences by race/ethnicity among patients not using a statin.

**Table 4:** Prevalence Ratios for LDL-C <70 mg/dL Associated With Statin Use, Sex, and Race/Ethnicity (N = 23,558)

	LDL-C <70 mg/dL, n (%)	Prevalence Ratio (95% CI)		
		Model 1	Model 2	Model 3^a^
Overall	10,028 (42.6)	–	–	–
By statin use				
Using a statin	8,180 (50.2)	1.97 (1.88-2.08)	1.93 (1.84-2.03)	1.96 (1.84-2.08)
Not using a statin	1,848 (25.4)	1.00 (ref)	1.00 (ref)	1.00 (ref)
By sex				
Using a statin				
Men	6,059 (51.7)	1.00 (ref)	1.00 (ref)	1.00 (ref)
Women	2,120 (46.5)	0.90 (0.87-0.93)	0.89 (0.86-0.92)	0.85 (0.81-0.89)
Not using a statin				
Men	1,375 (26.5)	1.00 (ref)	1.00 (ref)	1.00 (ref)
Women	472 (22.9)	0.86 (0.79-0.95)	0.81 (0.74-0.90)	0.79 (0.70-0.89)
By race/ethnicity				
Using a statin				
Non-Hispanic White	6,087 (53.8)	1.00 (ref)	1.00 (ref)	1.00 (ref)
Non-Hispanic Black	811 (34.9)	0.65 (0.61-0.69)	0.72 (0.67-0.77)	0.70 (0.65-0.75)
Hispanic	691 (46.1)	0.86 (0.81-0.91)	0.92 (0.87-0.98)	0.91 (0.84-0.99)
Other	375 (52.9)	0.98 (0.91-1.06)	1.02 (0.94-1.10)	0.96 (0.87-1.07)
Not using a statin				
Non-Hispanic White	1,357 (26.4)	1.00 (ref)	1.00 (ref)	1.00 (ref)
Non-Hispanic Black	184 (20.9)	0.79 (0.68-0.91)	0.91 (0.79-1.06)	0.94 (0.77-1.15)
Hispanic	155 (23.7)	0.90 (0.76-1.05)	0.95 (0.81-1.11)	1.03 (0.85-1.25)
Other	75 (24.9)	0.94 (0.77-1.15)	0.97 (0.79-1.20)	0.99 (0.79-1.25)

Model 1 is unadjusted.

Model 2 includes sex and race/ethnicity, simultaneously, with adjustment for calendar year, age, and geographic region.

Model 3 includes sex and race/ethnicity, with adjustment for calendar year, age, geographic region, payment source, unmet social needs, body mass index, history of smoking, stroke, PAD, heart failure, and cancer, prior CABG and PCI, diabetes, dyslipidemia, hypertension, and hospital-level characteristics, including bed size, accreditation by The Joint Commission, adult cardiac surgery, adult cardiology services, adult diagnostic catheterization, adult interventional cardiac catheterization, rural referral center, sole community provider, teaching status, and interaction terms between sex and race/ethnicity to determine whether sex disparities vary across racial/ethnic groups. Model 3 also includes prior use of ezetimibe and PCSK9i.

Abbreviation as in Table 1.

a *P* value for the interaction between sex and racial/ethnic groups among patients using a statin: 0.16. *P* value for the interaction between sex and racial/ethnic groups among patients not using a statin: 0.64.

In secondary analyses, 67.7% of patients had LDL-C <100 mg/dL, including 75.9% and 49.4% among those using and not using a statin, respectively (Supplemental Table 4). Also, 25.4% of patients had LDL-C <55 mg/dL, including 30.4% and 14.4% among those using and not using a statin, respectively (Supplemental Table 5). After full multivariable adjustment, women were less likely to have LDL-C <55 mg/dL, both among patients using a statin (PR: 0.83; 95% CI: 0.77-0.89) and not using a statin (PR: 0.75; 95% CI 0.64-0.89). Similarly, compared with non-Hispanic White patients, non-Hispanic Black patients were less likely to have an LDL-C <55 mg/dL among those using a statin (PR: 0.65; 95% CI: 0.57-0.74) and not using a statin (PR: 0.70; 95% CI: 0.51-0.96).

### Exploratory analyses

After full multivariable adjustment, the PR for statin use associated with uninsured status was 0.83 (95% CI: 0.78-0.89), whereas the PR associated with dyslipidemia was 1.37 (95% CI: 1.32-1.42). All other adjusted PRs ranged between 0.90 and 1.13 (Table 5). After full multivariable adjustment, older age, PCSK9i use, and diabetes were associated with a higher likelihood of LDL-C <70 mg/dL, whereas uninsured status and undocumented payment source were associated with a lower likelihood of LDL-C <70 mg/dL (Supplemental Table 6). Few patients with LDL-C <70 mg/dL or ≥70 mg/dL received combination lipid-lowering therapy (Supplemental Table 7).

**Table 5:** Patient Characteristics Associated With Statin Use at Admission (N = 34,003). Exploratory Analysis

	Statin Use, n (%)	Prevalence Ratio (95% CI)	
		Unadjusted	Fully Adjusted
Year			
2023	11,257 (68.4)	1.00 (ref)	1.00 (ref)
2024	12,008 (68.4)	1.00 (0.98-1.02)	1.00 (0.98-1.02)
Age			
18 to 44 years	549 (56.5)	0.89 (0.83-0.94)	0.95 (0.90-1.02)
45 to 59 years	4,417 (63.8)	1.00 (ref)	1.00 (ref)
60 to 69 years	6,555 (67.6)	1.06 (1.03-1.08)	1.01 (0.98-1.03)
70 to 79 years	7,102 (71.6)	1.12 (1.10-1.15)	1.03 (1.00-1.06)
≥80 years	4,642 (71.4)	1.12 (1.09-1.15)	1.03 (0.99-1.06)
Geographic region			
Eastern	5,833 (73.6)	1.00 (ref)	1.00 (ref)
Midwest	3,902 (68.0)	0.92 (0.87-0.98)	0.94 (0.89-1.00)
Western	3,510 (66.6)	0.90 (0.86-0.95)	0.95 (0.89-1.01)
Southeast	4,113 (65.1)	0.88 (0.84-0.93)	0.95 (0.90-0.99)
Southwest	5,901 (67.6)	0.92 (0.88-0.96)	0.95 (0.91-1.00)
Payment source			
Traditional Medicare	3,096 (66.9)	1.00 (ref)	1.00 (ref)
Medicare Advantage	4,904 (72.0)	1.08 (1.03-1.12)	1.04 (1.00-1.08)
Other government insurance	5,143 (69.8)	1.04 (1.00-1.09)	1.04 (0.99-1.08)
Private	4,583 (66.1)	0.99 (0.95-1.03)	1.02 (0.98-1.06)
Government and private	4,000 (73.3)	1.10 (1.05-1.14)	1.01 (0.98-1.05)
Uninsured	798 (50.1)	0.75 (0.69-0.81)	0.83 (0.78-0.89)
Not documented	176 (54.5)	0.81 (0.71-0.94)	0.94 (0.82-1.08)
Unmet social needs			
Yes	1,497 (64.9)	0.90 (0.86-0.94)	0.94 (0.90-0.98)
No	12,630 (71.8)	1.00 (ref)	1.00 (ref)
Not assessed	9,138 (64.8)	0.90 (0.87-0.94)	0.94 (0.91-0.97)
Body mass index			
<18.5 kg/m^2^	227 (65.4)	0.97 (0.90-1.05)	0.98 (0.90-1.07)
18.5 to <25 kg/m^2^	5,036 (67.2)	1.00 (ref)	1.00 (ref)
25 to < 30 kg/m^2^	8,117 (68.6)	1.02 (1.00-1.04)	1.00 (0.98-1.02)
≥30 Kg/m2	9,301 (69.7)	1.04 (1.02-1.06)	1.01 (0.99-1.03)
History of smoking	7,444 (64.7)	0.92 (0.90-0.94)	0.97 (0.95-0.99)
No history of smoking	15,773 (70.5)	1.00 (ref)	1.00 (ref)
History of stroke	2,845 (73.7)	1.09 (1.06-1.11)	1.03 (1.01-1.06)
No history of stroke	20,420 (67.7)	1.00 (ref)	1.00 (ref)
History of PAD	2,699 (77.8)	1.15 (1.13-1.18)	1.04 (1.01-1.06)
No history of PAD	20,566 (67.4)	1.00 (ref)	1.00 (ref)
History of heart failure	6,594 (76.4)	1.16 (1.14-1.18)	1.09 (1.07-1.11)
No history of heart failure	16,671 (65.7)	1.00 (ref)	1.00(ref)
History of cancer	2,746 (74.2)	1.10 (1.07-1.12)	1.04 (1.01-1.06)
No history of cancer	20,519 (67.7)	1.00 (ref)	1.00 (ref)
Prior CABG	6,803 (73.6)	1.11 (1.09-1.12)	1.07 (1.05-1.09)
No prior CABG	16,462 (66.5)	1.00 (ref)	1.00 (ref)
Prior PCI	17,350 (70.5)	1.12 (1.10-1.14)	1.08 (1.06-1.11)
No prior PCI	5,915 (62.9)	1.00 (ref)	1.00 (ref)
Diabetes	11,788 (74.5)	1.18 (1.16-1.20)	1.10 (1.08-1.12)
Without diabetes	11,477 (63.1)	1.00 (ref)	1.00 (ref)
Dyslipidemia	19,485 (74.0)	1.50 (1.45-1.56)	1.37 (1.32-1.42)
Without dyslipidemia	3,780 (49.2)	1.00 (ref)	1.00 (ref)
Hypertension	20,924 (70.9)	1.36 (1.31-1.41)	1.13 (1.09-1.18)
Without hypertension	2,341 (52.0)	1.00 (ref)	1.00 (ref)
Hospital-level characteristics			
Bed size			
Less than 100 beds	1,621 (56.2)	0.81 (0.73-0.89)	0.91 (0.81-1.02)
100-199 beds	4,746 (67.4)	0.97 (0.92-1.03)	0.99 (0.95-1.04)
200-299 beds	5,456 (69.5)	1.00 (ref)	1.00 (ref)
300-399 beds	2,878 (69.9)	1.01 (0.94-1.08)	1.01 (0.94-1.08)
400-499 beds	1,940 (68.7)	0.99 (0.92-1.06)	1.01 (0.94-1.08)
500 or more beds	5,993 (71.6)	1.03 (0.97-1.09)	1.01 (0.95-1.07)
Accreditation by The Joint Commission			
Yes	18,797 (68.8)	1.03 (0.98-1.08)	0.99 (0.95-1.04)
No	3,837 (66.8)	1.00 (ref)	1.00 (ref)
Adult cardiac surgery			
Yes	14,459 (70.2)	1.09 (1.04-1.14)	1.02 (0.97-1.07)
No	5,229 (64.5)	1.00 (ref)	1.00 (ref)
Adult cardiology services			
Yes	18,900 (69.1)	1.19 (1.07-1.33)	0.90 (0.76-1.07)
No	788 (57.9)	1.00 (ref)	1.00 (ref)
Adult diagnostic catheterization			
Yes	18,614 (69.5)	1.25 (1.10-1.42)	0.94 (0.83-1.07)
No	1,074 (55.6)	1.00 (ref)	1.00 (ref)
Adult interventional cardiac catheterization			
Yes	18,589 (69.6)	1.27 (1.13-1.43)	1.12 (0.98-1.29)
No	1,099 (54.9)	1.00 (ref)	1.00 (ref)
Rural referral center			
Yes	7,923 (71.4)	1.07 (1.03-1.11)	1.03 (1.00-1.07)
No	14,711 (66.9)	1.00 (ref)	1.00 (ref)
Sole community provider			
Yes	667 (66.7)	0.97 (0.92-1.03)	1.11 (1.02-1.21)
No	21,967 (68.5)	1.00 (ref)	1.00 (ref)
Teaching status^a^			
Major	3,770 (72.6)	1.16 (1.09-1.24)	1.04 (0.97-1.12)
Minor	14,425 (69.4)	1.11 (1.05-1.18)	1.02 (0.97-1.08)
Nonteaching	4,439 (62.5)	1.00 (ref)	1.00 (ref)

The unadjusted model includes each variable one at a time.

The fully adjusted model includes all variables in the table, simultaneously, and sex, race/ethnicity, and interaction terms between sex and race/ethnicity.

Abbreviations as in Table 1.

a Teaching status: major teaching hospitals - those with Council of Teaching Hospitals designation (COTH); minor teaching hospitals - those approved to participate in residency and/or internship training by the Accreditation Council for Graduate Medical Education (ACGME), or the American Osteopathic Association (AOA); or those with medical school affiliation reported to the American Medical Association (AMA); nonteaching hospitals - those without COTH, ACGME, AOA or Medical School (AMA) affiliation.

## Discussion

In this analysis of patients with CAD hospitalized for an acute coronary event, only two-thirds were using a statin (Central Illustration). Most patients had LDL-C levels above those recommended in the 2018 and 2026 joint multisociety cholesterol guidelines.^1^^,^^2^ Statin use was modestly less common among women. Women and non-Hispanic Black and Hispanic patients were less likely to have LDL-C <70 mg/dL, particularly among those receiving statins. The low use of statin therapy at admission contrasted markedly with the frequent prescription of high-intensity statin therapy at discharge. The current results indicate substantial underuse of guideline-recommended lipid-lowering therapy in the ambulatory setting and a missed opportunity to reduce the burden of coronary events in this high-risk population.

**Central Illustration:**
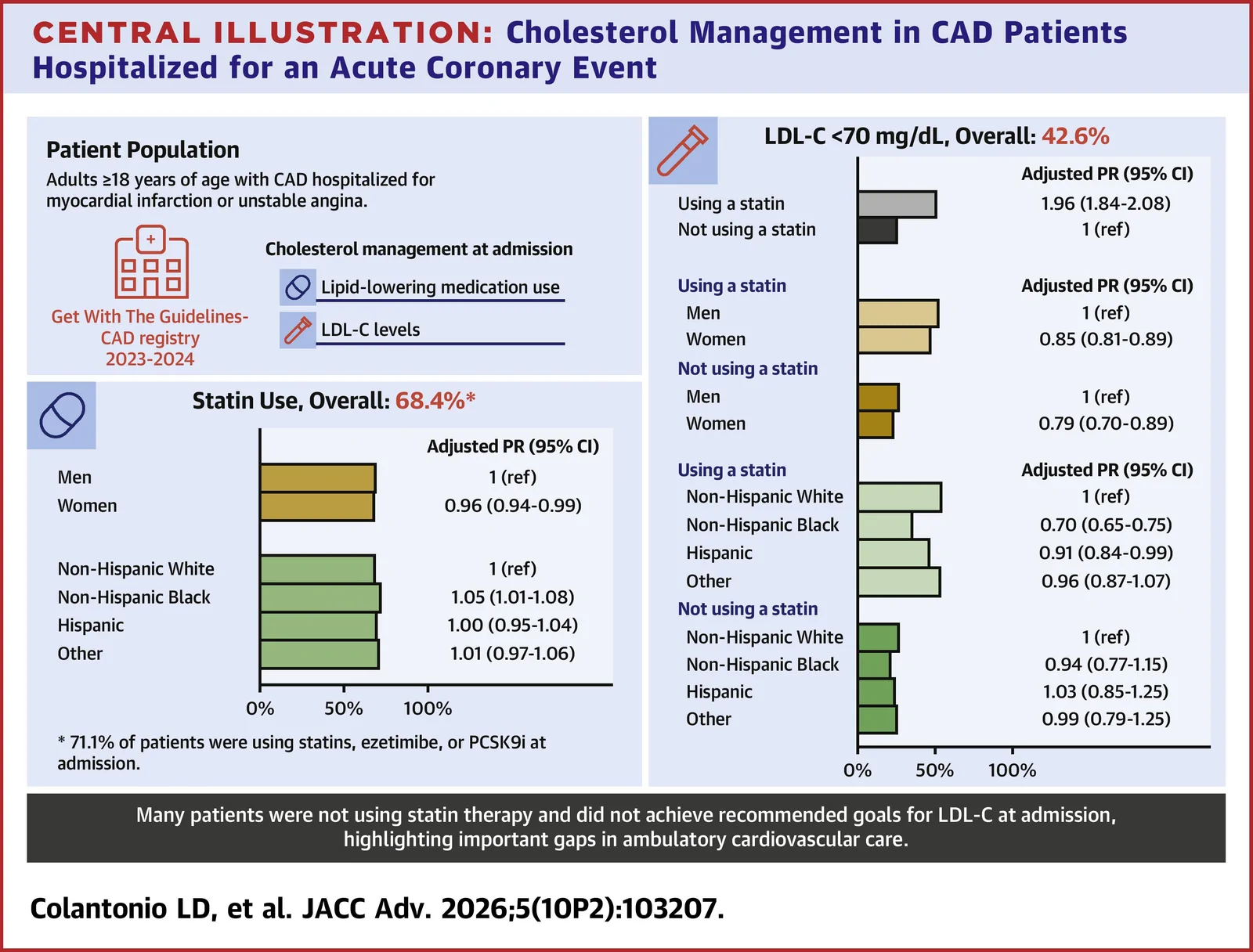
Cholesterol Management in CAD Patients Hospitalized for an Acute Coronary Event Icons incorporated into this illustration were obtained from SVG Repo (https://www.svgrepo.com), including hospital icon by IonutNeagu (Public Domain), blood sample icon by SVG Repo (CC0), and pill icon by Solar Icons (CC Attribution License). CAD = coronary artery disease; LDL-C = low-density lipoprotein cholesterol; PCSK9i = proprotein convertase subtilisin/kexin type 9 inhibitor; PR = prevalence ratio.

Using NHANES data, it has been estimated that 67.9% of all U.S. adults with CAD used a statin in 2015 to 2020.^7^ Also, only 30.3% and 17.4% of U.S. adults with a prior myocardial infarction in 2015 to 2023 had an LDL-C <70 mg/dL and <55 mg/dL, respectively.^21^ In 2017, 80.1% of ambulatory patients with CAD in the ACC PINNACLE registry were using a statin.^22^ In 2021, 76.1% of ambulatory patients with cardiovascular disease in the cvMOBIUS2 registry were using a statin, and 44.5% had an LDL-C <70 mg/dL.^23^ Institutions participating in the PINNACLE and cvMOBIUS2 registries may have high standards of care, which may explain the better cholesterol management compared with NHANES. Institutions participating in the GWTG-CAD may also have high standards of care. However, the population with an acute coronary event in the GWTG-CAD registry may be enriched by patients with poor cholesterol management (versus all individuals with CAD). This may explain the lower prevalence of statin use and LDL-C <70 mg/dL in the current analysis compared with the PINNACLE and cvMOBIUS2 registries.

Better cholesterol management is known to prevent coronary events.^1^^,^^24^ In the current analysis, ∼10,000 patients with CAD hospitalized for an acute coronary event were not using a statin (mean LDL-C ∼100 mg/dL). Based on prior clinical trials, it can be estimated that, had these patients been receiving high-intensity statin therapy before admission, their LDL-C levels may have been up to 50% lower (50 mg/dL or ∼1.28 mmol/L), potentially translating into a 30% relative risk reduction or 3,000 fewer hospitalizations.^1^^,^^24^ Data on the intensity of statin therapy at admission are unavailable in the GWTG-CAD registry; however, only half of patients receiving a statin in the cvMOBIUS2 and GOULD registries were using a high-intensity dosage.^23^^,^^25^ Intensification to maximally tolerated dosage could have further reduced the risk of coronary events among many statin users in the current study.^26^ Adding ezetimibe or a PCSK9i can also help to achieve LDL-C goals and reduce the risk for CAD events.^1^^,^^2^ In a prior simulation of patients with a myocardial infarction (77.5% using a statin, mean LDL-C 92.6 mg/dL) using evidence from clinical trials, initiation or intensification of statin therapy with the addition of ezetimibe or a PCSK9i when needed to achieve an LDL-C <70 mg/dL and <55 mg/dL was estimated to reduce the risk for recurrent CAD events by 24.3% and 27.6%, respectively.^27^ In the current study, few patients used combination lipid-lowering therapies. Greater use of combination lipid-lowering therapy can improve cholesterol management and reduce coronary events.^2^

Over the past 25 years, inpatient quality improvement initiatives have helped improve cholesterol management following a CAD hospitalization.^5^^,^^6^^,^^8^ However, persistent ambulatory management gaps underscore the need for outpatient quality improvement interventions. Patient and provider education programs, institutional lipid management protocols, and follow-up by a cardiologist could reduce ambulatory cholesterol management gaps.^25^^,^^28^, ^29^, ^30^ Prior studies have shown that LDL-C testing is underutilized in patients with CAD.^25^^,^^31^ Increasing LDL-C monitoring may help to break clinical inertia and reduce management gaps.^23^^,^^29^ Initiated in 2021, the AHA National Integrated ASCVD Initiative is a quality improvement program designed to optimize LDL-C monitoring and management in ambulatory patients with ASCVD by improving inpatient-outpatient coordination.^32^ The proportion of participants in this initiative who had an LDL-C <70 mg/dL increased from 48.4% in 2022 to 49.5% in 2023 (*P* value <0.005).

The current results suggest the persistence of sex and racial/ethnic disparities in cholesterol management.^9^, ^10^, ^11^, ^12^, ^13^ The lower prevalence of statin use and LDL-C <70 mg/dL observed in women compared with men likely involves multiple patient- and provider-level factors, including higher frequency of statin-related symptoms and lower perceived need for this therapy among women.^10^ Lower use of statin therapy in non-Hispanic Black vs non-Hispanic White U.S. patients has been previously reported, possibly due to unmeasured structural factors, including bias, stereotyping, or mistrust.^12^^,^^13^ In the current study, LDL-C <70 mg/dL was less common in non-Hispanic Black and Hispanic patients using a statin compared to their non-Hispanic White counterparts. However, statin use was higher among non-Hispanic Black vs non-Hispanic White patients. A potential explanation for racial differences in statin use observed in the current analysis is the selection of individuals with a hospitalization rather than the entire CAD population. In the REasons for Geographic And Racial Differences in Stroke (REGARDS) study, Black participants who discontinued statin were more willing to reinitiate this therapy compared to their White counterparts (age-sex-adjusted PR: 1.29; 95% CI: 1.05-1.59).^33^ In addition, Black men were less likely to experience nonfatal myocardial infarction and more likely to have fatal coronary events than White men, potentially due to the underdetection or delayed recognition of symptoms among the former.^34^ By selecting individuals with an acute coronary hospitalization rather than all CAD patients, the current analysis may include an over-representation of non-Hispanic Black patients with more proactive health-seeking behaviors, including both early recognition of coronary symptoms and high willingness to use statins (ie, collider stratification bias).^35^

Most patient characteristics in the current exploratory analyses showed a small association with statin use and LDL-C, indicating that treatment gaps are not limited to specific subpopulations. After multivariable adjustment, being uninsured was associated with a 17% lower likelihood of statin use and a 24% lower likelihood of LDL-C <70 mg/dL. Lowering economic barriers may improve statin adherence and reduce the risk for CAD events.^36^^,^^37^ Diabetes was associated with a higher likelihood of statin use, potentially due to a higher perceived need for statins in this population. Prior studies in the cvMOBIUS2, GOULD, and PALM registries have consistently shown better cholesterol management in patients with diabetes.^23^^,^^25^^,^^28^ Reasons for better cholesterol management in people with diabetes may be multifactorial, including patient-related (eg, treatment adherence) and provider-related factors (eg, more frequent cholesterol monitoring).^38^^,^^39^ LDL-C <70 mg/dL was more common in older adults, which may reflect a higher frequency of catabolic states, frailty, or malnutrition.^40^

### Strengths and limitations

The current analysis used standardized data from a large sample of patients and hospitals from all U.S. census regions. However, this study has limitations. The GWTG program is a quality improvement initiative. Participating hospitals likely differ from nonparticipating hospitals in many aspects, including patient and provider demographics (eg, race/ethnicity), resources focused on improvement in cardiovascular care, and integration of inpatient and outpatient care, potentially limiting generalizability of our findings. Data on how long patients were not using a statin and their reasons (eg, statin intolerance) were unavailable. However, most patients who were discharged alive to their homes were prescribed this medication. Data on myocardial infarction subtype (type I vs type II) were not available in the GWTG-CAD registry. Because the current analysis included CAD patients hospitalized for an acute coronary event, selection of those with worse cholesterol management and collider stratification bias cannot be excluded. Therefore, the results may not be generalizable to all patients with CAD. Some GWTG registry variables, including LDL-C at admission, are optional, and noncompletion may be related to administrative or documentation burden. Consequently, current findings may not be generalizable to patients without documented LDL-C at admission. Given the large sample size, some statistically significant associations were modest in magnitude and may have limited clinical relevance.

## Conclusions

Among patients with known CAD hospitalized with an acute coronary event, prior cholesterol management is frequently suboptimal. This represents a missed opportunity for outcomes prevention, imposing a substantial burden on patients and health systems. Targeted quality improvement strategies are needed to address persistent cholesterol management gaps in the ambulatory setting and improve secondary prevention outcomes.

Perspectives **COMPETENCY IN PATIENT CARE AND PROCEDURAL SKILLS:** Among patients with established coronary artery disease hospitalized for myocardial infarction or unstable angina, substantial gaps in outpatient cholesterol management remain. Approximately one-third were not receiving statin therapy before hospitalization, and fewer than half achieved guideline-recommended LDL-C levels below 70 mg/dL. These findings highlight the need for clinicians to routinely assess adherence, optimize statin treatment, and consider intensification with additional lipid-lowering therapies when indicated. Women and Black patients experienced lower rates of LDL-C goal attainment, underscoring the importance of equitable, patient-centered approaches to secondary prevention.

**COMPETENCY IN SYSTEMS-BASED PRACTICE:** Hospitalization for an acute coronary event represents a critical opportunity to identify failures in ambulatory cardiovascular prevention and strengthen transitions of care. Health systems, quality improvement programs, and multidisciplinary care teams should implement strategies that support ambulatory lipid monitoring, treatment intensification, medication access, and long-term follow-up for patients with established CAD. Addressing these gaps may reduce recurrent cardiovascular events and associated healthcare utilization and costs.

**TRANSLATIONAL OUTLOOK:** Substantial opportunities exist to improve cholesterol management among patients with established coronary artery disease. Health systems and clinicians should prioritize systematic approaches to identify ambulatory patients with uncontrolled LDL-C, optimize statin therapy, intensify treatment with non-statin lipid-lowering agents when appropriate, and address barriers to medication adherence and access. Targeted interventions focused on populations with lower rates of LDL-C goal attainment, including women and Black patients, may help reduce disparities and improve secondary prevention outcomes.

## Funding support and author disclosures

This study was funded by the American Heart Association grant “Statin use and LDL-C levels in patients with a history of CAD who are hospitalized for an acute coronary syndrome” (25GWTGDRASp1454022) to Dr Colantonio. Dr Colantonio has received research grant support from Amgen. Levitan received research grant from Amgen (past); consulting fees from Pfizer; and honorarium as Data Safety Monitoring Board member from the University of Pittsburgh. Dr Bittner has received research grants to her institution from Amgen (EVOLVE MI, Site PI), Novartis (ORION IV trial, Site PI), and Atrium Health/Wake Forest (RehabHFpEF); has received payments as a Data Safety Monitoring Board member (Eli Lilly; Verve Therapeutics); and has received consulting fees from New Amsterdam Pharma (2023). All other authors have reported that they have no relationships relevant to the contents of this paper to disclose.
