# Supplementary material for: Cholesterol Management Gaps Among Patients With Established Coronary Artery Disease Hospitalized for Acute Coronary Events
Source: JACC Adv. 2026 Sep 3;5(10):103207. doi: 10.1016/j.jacadv.2026.103207 (PMC13571717; doi:10.1016/j.jacadv.2026.103207)
Supplement: Supplementary file 1 [file mmc1.docx]

**Cholesterol Management Gaps Among Patients with Established Coronary Artery Disease Hospitalized for Acute Coronary Events.**

**Cross-sectional analysis of the GWTG-CAD registry.**

**SUPPLEMENTAL MATERIAL**

Lisandro D. Colantonio,^a^ MD, PhD, Zhixin Wang,^a^ MS, Stephen L. Sigal,^b^ MD, MS, Emily B. Levitan,^a^ ScD, Vera Bittner,^c^ MD, MSPH.

a. Department of Epidemiology, University of Alabama at Birmingham, Birmingham, AL, US.
b. Heart and Vascular Institute at Titus, Mt. Pleasant, TX, US.
c. Department of Medicine, Division of Cardiovascular Disease, University of Alabama at Birmingham, Birmingham, AL, US.

**Corresponding Author:**

Lisandro D. Colantonio. 1720 2nd Ave South, RPHB 527C. Birmingham, AL 35294-0013. Phone: 205-259-6415. Fax: 205-975-7058. Email: [lcolantonio@uab.edu](mailto:lcolantonio@uab.edu). ORCID: 0000-0001-8742-1788. X Handle: @lcolantonio.

**Supplemental Table 1.** Prevalence ratios for using a statin, ezetimibe or PCSK9i at admission (n=34,003). Sensitivity analysis.

|  |  | Prevalence ratio (95% CI) | | |
| --- | --- | --- | --- | --- |
|  | Patients using a statin, ezetimibe or PCSK9i, n (%) | Model 1 | Model 2 | Model 3* |
| Overall | 24,167 (71.1) | --- | --- | --- |
| **Sex** |  |  |  |  |
| Men | 17,151 (71.0) | 1 (ref) | 1 (ref) | 1 (ref) |
| Women | 7,011 (71.3) | 1.00 (0.99-1.02) | 0.99 (0.97-1.00) | 0.99 (0.97-1.01) |
| **Race/ethnicity** |  |  |  |  |
| Non-Hispanic white | 17,044 (71.1) | 1 (ref) | 1 (ref) | 1 (ref) |
| Non-Hispanic Black | 3,325 (73.8) | 1.04 (1.01-1.07) | 1.06 (1.03-1.09) | 1.03 (1.00-1.07) |
| Hispanic | 2,066 (70.5) | 0.99 (0.96-1.03) | 1.02 (0.98-1.05) | 0.99 (0.94-1.03) |
| Other | 1,044 (72.1) | 1.01 (0.97-1.06) | 1.03 (0.99-1.07) | 1.01 (0.97-1.05) |
| BMI: body mass index; CABG: coronary artery bypass grafting; CI: confidence interval; LDL-C: low-density lipoprotein cholesterol; PAD: peripheral artery disease; PCI: percutaneous coronary intervention; PCSK9i: Proprotein Convertase Subtilisin/Kexin Type 9 inhibitor.  Model 1 is unadjusted.  Model 2 includes sex and race/ethnicity, simultaneously, with adjustment for calendar year, age, and geographic region.  Model 3 includes sex and race/ethnicity, with adjustment for calendar year, age, geographic region, payment source, unmet social needs, BMI, history of smoking, stroke, PAD, heart failure, and cancer, prior CABG and PCI, diabetes, dyslipidemia, hypertension, and hospital-level characteristics, including bed size, accreditation by The Joint Commission, adult cardiac surgery, adult cardiology services, adult diagnostic catheterization, adult interventional cardiac catheterization, rural referral center, sole community provider, and teaching Status. Model 3 also includes interaction terms between sex and racial/ethnic groups to determine whether sex disparities differ by race/ethnicity.  * p-value for the interaction between sex and racial/ethnic groups = 0.75. | | | | |

**Supplemental Table 2**. Statin prescription among patients who were discharged alive to their homes by sex and race/ethnicity (n=25,062).

|  |  | Prevalence ratio (95% CI) | | | |
| --- | --- | --- | --- | --- | --- |
|  | n (%) | Model 1 | Model 2 | Model 3* | |
| **Prescribed a statin** | | | | | |
| Men | 17,076 (94.6) | 1 (ref) | 1 (ref) | 1 (ref) | |
| Women | 6,379 (91.0) | 0.96 (0.95-0.97) | 0.96 (0.96-0.97) | 0.95 (0.94-0.97) | |
| Non-Hispanic white | 16,150 (92.6) | 1 (ref) | 1 (ref) | 1 (ref) | |
| Non-Hispanic Black | 3,271 (95.9) | 1.04 (1.03-1.04) | 1.03 (1.03-1.04) | 1.02 (1.01-1.03) | |
| Hispanic | 2,245 (96.4) | 1.04 (1.03-1.05) | 1.04 (1.03-1.05) | 1.03 (1.02-1.04) | |
| Other | 1,061 (96.6) | 1.04 (1.03-1.06) | 1.04 (1.02-1.05) | 1.01 (1.00-1.03) | |
| **Prescribed high-intensity statin therapy** | | | | | |
| Men | 15,684 (86.9) | 1 (ref) | 1 (ref) | 1 (ref) | |
| Women | 5,699 (81.3) | 0.94 (0.92-0.95) | 0.94 (0.93-0.96) | 0.93 (0.91-0.95) | |
| Non-Hispanic white | 14,526 (83.3) | 1 (ref) | 1 (ref) | 1 (ref) | |
| Non-Hispanic Black | 3,094 (90.7) | 1.09 (1.07-1.10) | 1.07 (1.05-1.08) | 1.04 (1.02-1.05) | |
| Hispanic | 2,102 (90.3) | 1.08 (1.06-1.10) | 1.07 (1.05-1.09) | 1.05 (1.03-1.07) | |
| Other | 975 (88.8) | 1.07 (1.04-1.10) | 1.05 (1.02-1.08) | 1.04 (1.00-1.07) | |
| CABG: coronary artery bypass grafting; CI: confidence interval; LDL-C: low-density lipoprotein cholesterol; PAD: peripheral artery disease; PCI: percutaneous coronary intervention.  Model 1 is unadjusted.  Model 2 includes sex and race/ethnicity, simultaneously, with adjustment for calendar year, age, and geographic region.  Model 3 includes sex and race/ethnicity, with adjustment for calendar year, age, geographic region, payment source, unmet social needs, body mass index, history of smoking, stroke, PAD, heart failure, and cancer, prior CABG and PCI, diabetes, dyslipidemia, hypertension, and hospital-level characteristics, including bed size, accreditation by The Joint Commission, adult cardiac surgery, adult cardiology services, adult diagnostic catheterization, adult interventional cardiac catheterization, rural referral center, sole community provider, and teaching Status. Model 3 also includes interaction terms between sex and racial/ethnic groups to determine whether sex disparities differ by race/ethnicity.  * p-value for the interaction between sex and racial/ethnic groups for statin nonprescription: 0.002. p-value for the interaction between sex and racial/ethnic groups for nonprescription of high-intensity statin therapy: 0.02. | | | | |  |

**Supplemental Table 3**. Characteristics of patients with LDL-C not documented at arrival.

|  | LDL-C documented  N=23,558 | | LDL-C not documented  n=10,445 |
| --- | --- | --- | --- |
|  | LDL-C < 70 mg/dL  n=10,028 | LDL-C ≥ 70 mg/dL  n=13,530 |  |
| Women, n (%) | 2,592 (25.9) | 4,033 (29.8) | 3,210 (30.7) |
| Race/ethnicity, n (%) |  |  |  |
| Non-Hispanic white | 7,444 (76.5) | 9,002 (68.8) | 7,540 (75.0) |
| Non-Hispanic Black | 995 (10.2) | 2,208 (16.9) | 1,305 (13.0) |
| Hispanic | 846 (8.7) | 1,307 (10.0) | 776 (7.7) |
| Other* | 450 (4.6) | 560 (4.3) | 439 (4.4) |
| Year, n (%) |  |  |  |
| 2023 | 4,775 (47.6) | 6,631 (49.0) | 5,040 (48.3) |
| 2024 | 5,253 (52.4) | 6,899 (51.0) | 5,405 (51.7) |
| Age, mean (SD) | 70.8 (11.1) | 66.0 (12.2) | 69.4 (12.3) |
| Age, n (%) |  |  |  |
| 18 to 44 years | 142 (1.4) | 548 (4.1) | 281 (2.7) |
| 45 to 59 years | 1,435 (14.3) | 3,535 (26.1) | 1,949 (18.7) |
| 60 to 69 years | 2,737 (27.3) | 4,144 (30.6) | 2,814 (26.9) |
| 70 to 79 years | 3,465 (34.6) | 3,366 (24.9) | 3,090 (29.6) |
| ≥80 years | 2,249 (22.4) | 1,937 (14.3) | 2,311 (22.1) |
| Geographic region, n (%) |  |  |  |
| Eastern | 2,489 (24.8) | 3,342 (24.7) | 2,093 (20.0) |
| Midwest | 1,638 (16.3) | 2,024 (15.0) | 2,077 (19.9) |
| Western | 1,632 (16.3) | 1,982 (14.7) | 1,659 (15.9) |
| Southeast | 1,879 (18.7) | 2,761 (20.4) | 1,682 (16.1) |
| Southwest | 2,387 (23.8) | 3,410 (25.2) | 2,929 (28.1) |
| Payment source, n (%) |  |  |  |
| Traditional Medicare | 1,380 (14.1) | 1,625 (12.2) | 1,625 (16.2) |
| Medicare Advantage | 2,312 (23.6) | 2,473 (18.6) | 2,022 (20.2) |
| Other government insurance† | 2,015 (20.5) | 2,993 (22.6) | 2,355 (23.5) |
| Private‡ | 1,814 (18.5) | 3,235 (24.4) | 1,883 (18.8) |
| Government and private† | 1,959 (20.0) | 1,902 (14.3) | 1,596 (15.9) |
| Uninsured | 280 (2.9) | 907 (6.8) | 407 (4.1) |
| Not documented | 48 (0.5) | 135 (1.0) | 140 (1.4) |
| Unmet social needs, n (%) |  |  |  |
| Yes | 560 (5.6) | 1,129 (8.3) | 619 (5.9) |
| No | 5,888 (58.7) | 7,395 (54.7) | 4,307 (41.2) |
| Not assessed | 3,580 (35.7) | 5,006 (37.0) | 5,519 (52.8) |
| Body mass index, n (%) |  |  |  |
| <18.5 Kg/m^2^ | 93 (0.9) | 128 (1.0) | 126 (1.3) |
| 18.5 to <25 Kg/m^2^ | 2,286 (23.2) | 2,904 (21.9) | 2,307 (23.3) |
| 25 to < 30 Kg/m^2^ | 3,622 (36.8) | 4,704 (35.4) | 3,508 (35.4) |
| ≥ 30 Kg/m^2^ | 3,836 (39.0) | 5,541 (41.7) | 3,971 (40.1) |
| History of smoking, n (%)§ | 2,724 (27.2) | 5,145 (38.1) | 3,644 (35.1) |
| History of stroke, n (%) | 1,185 (11.8) | 1,464 (10.8) | 1,212 (11.6) |
| History of PAD, n (%) | 1,126 (11.2) | 1,236 (9.1) | 1,108 (10.6) |
| History of heart failure, n (%) | 2,653 (26.5) | 3,059 (22.6) | 2,923 (28.0) |
| History of cancer, n (%) | 1,321 (13.2) | 1,214 (9.0) | 1,165 (11.2) |
| Prior CABG, n (%) | 3,016 (30.1) | 3,204 (23.7) | 3,025 (29.0) |
| Prior PCI, n (%) | 7,490 (74.7) | 9,980 (73.8) | 7,135 (68.3) |
| Diabetes, n (%) | 5,130 (51.2) | 5,764 (42.6) | 4,933 (47.2) |
| Dyslipidemia, n (%)∥ | 8,293 (82.7) | 10,540 (77.9) | 7,488 (71.7) |
| Hypertension, n (%) | 9,004 (89.8) | 11,607 (85.8) | 8,890 (85.1) |
| Using a statin, n (%) | 8,180 (81.6) | 8,114 (60.0) | 6,971 (66.7) |
| Using ezetimibe, n (%) | 957 (9.5) | 916 (6.8) | 675 (6.5) |
| Using PCSK9i, n (%) | 273 (2.7) | 205 (1.5) | 163 (1.6) |
| **Hospital-level characteristics** |  |  |  |
| Bed size (categories), n (%) |  |  |  |
| Less than 100 beds | 345 (3.5) | 498 (3.8) | 2,039 (20.1) |
| 100-199 beds | 2,147 (22.0) | 2,570 (19.5) | 2,320 (22.8) |
| 200-299 beds | 2,238 (22.9) | 3,177 (24.1) | 2,431 (23.9) |
| 300-399 beds | 1,338 (13.7) | 1,712 (13.0) | 1,066 (10.5) |
| 400-499 beds | 910 (9.3) | 1,218 (9.3) | 694 (6.8) |
| 500 or more beds | 2,781 (28.5) | 3,985 (30.3) | 1,609 (15.8) |
| Accreditation by The Joint Commission, n (%) | 8,287 (84.9) | 11,243 (85.4) | 7,807 (76.8) |
| Adult cardiac surgery, n (%) | 6,649 (77.5) | 8,975 (76.6) | 4,975 (59.2) |
| Adult cardiology services, n (%) | 8,458 (98.6) | 11,563 (98.7) | 7,321 (87.1) |
| Adult diagnostic catheterization, n (%) | 8,418 (98.1) | 11,515 (98.3) | 6,839 (81.3) |
| Adult interventional cardiac catheterization, n (%) | 8,427 (98.2) | 11,503 (98.2) | 6,770 (80.5) |
| Rural referral center, n (%) | 3,513 (36.0) | 4,567 (34.7) | 3,014 (29.7) |
| Sole community provider, n (%) | 214 (2.2) | 261 (2.0) | 525 (5.2) |
| Teaching status, n (%)¶ |  |  |  |
| Major | 1,732 (17.7) | 2,579 (19.6) | 881 (8.7) |
| Minor | 6,366 (65.2) | 8,290 (63.0) | 6,123 (60.3) |
| Non-teaching | 1,661 (17.0) | 2,291 (17.4) | 3,155 (31.1) |
| BMI: body mass index; CABG: coronary artery bypass grafting; LDL-C: low-density lipoprotein cholesterol; PAD: peripheral artery disease; PCI: percutaneous coronary intervention; PCSK9i: Proprotein Convertase Subtilisin/Kexin Type 9 inhibitor; SD: standard deviation.  * The other race category includes American Indian or Alaska Native, Asian, and Native Hawaiian or Pacific Islander.  † Government insurance includes Medicare, Medicaid, Veterans Affairs, Civilian Health and Medical Program of the Department of Veterans Affairs (CHAMPVA), Tricare, and Indian Health Services.  ‡ Private insurance includes commercial insurance (Not Medicare/ Medicaid) typically tied to employer-based plans.  § In the past 12 months.  ∥ Includes history of familial hypercholesterolemia.  ¶ Teaching status: Major Teaching Hospitals - those with Council of Teaching Hospitals designation (COTH); Minor Teaching Hospitals - those approved to participate in residency and/or internship training by the Accreditation Council for Graduate Medical Education (ACGME), or the American Osteopathic Association (AOA); or those with medical school affiliation reported to the American Medical Association (AMA); Non-Teaching Hospitals - those without COTH, ACGME, AOA or Medical School (AMA) affiliation. | | | |

**Supplemental Table 4.** Prevalence ratios for LDL-C <100 mg/dL at arrival associated with statin use, sex, and race/ethnicity, among those with LDL-C documented (n=23,558). Sensitivity analysis.

|  |  | Prevalence ratio (95% CI) | | | |
| --- | --- | --- | --- | --- | --- |
|  | LDL-C <100 mg/dL, n (%) | Model 1 | Model 2 | Model 3* | |
| Overall | 15,960 (67.7) | --- | --- | --- | |
| **By statin use** |  |  |  |  | |
| Using a statin | 12,369 (75.9) | 1.54 (1.49-1.58) | 1.51 (1.47-1.55) | 1.52 (1.47-1.57) | |
| Not using a statin | 3,591 (49.4) | 1 (ref) | 1 (ref) | 1 (ref) | |
| **By sex** |  |  |  |  | |
| **Using a statin** |  |  |  |  | |
| Men | 9,063 (77.3) | 1 (ref) | 1 (ref) | 1 (ref) | |
| Women | 3,303 (72.4) | 0.94 (0.92-0.96) | 0.93 (0.91-0.95) | 0.92 (0.90-0.94) | |
| **Not using a statin** |  |  |  |  | |
| Men | 2,645 (50.9) | 1 (ref) | 1 (ref) | 1 (ref) | |
| Women | 945 (45.8) | 0.90 (0.85-0.95) | 0.87 (0.82-0.92) | 0.81 (0.75-0.87) | |
| **By race/ethnicity** |  |  |  |  | |
| **Using a statin** |  |  |  |  | |
| Non-Hispanic white | 8,887 (78.6) | 1 (ref) | 1 (ref) | 1 (ref) | |
| Non-Hispanic Black | 1,512 (65.1) | 0.83 (0.80-0.86) | 0.88 (0.85-0.91) | 0.88 (0.85-0.92) | |
| Hispanic | 1,103 (73.6) | 0.94 (0.91-0.97) | 0.98 (0.95-1.01) | 0.98 (0.94-1.02) | |
| Other | 537 (75.7) | 0.96 (0.92-1.01) | 0.99 (0.94-1.04) | 0.96 (0.90-1.02) | |
| **Not using a statin** |  |  |  |  | |
| Non-Hispanic white | 2,557 (49.8) | 1 (ref) | 1 (ref) | 1 (ref) | |
| Non-Hispanic Black | 412 (46.8) | 0.94 (0.87-1.01) | 1.03 (0.95-1.11) | 1.00 (0.91-1.11) | |
| Hispanic | 324 (49.5) | 1.00 (0.91-1.09) | 1.03 (0.94-1.13) | 1.00 (0.87-1.15) | |
| Other | 158 (52.5) | 1.05 (0.94-1.18) | 1.10 (0.98-1.23) | 1.01 (0.88-1.16) | |
| BMI: body mass index; CABG: coronary artery bypass grafting; CI: confidence interval; LDL-C: low-density lipoprotein cholesterol; PAD: peripheral artery disease; PCI: percutaneous coronary intervention; PCSK9i: Proprotein Convertase Subtilisin/Kexin Type 9 inhibitor.  Model 1 is unadjusted.  Model 2 includes sex and race/ethnicity, simultaneously, with adjustment for calendar year, age, and geographic region.  Model 2 includes sex and race/ethnicity, with adjustment for calendar year, age, geographic region, payment source, unmet social needs, BMI, history of smoking, stroke, PAD, heart failure, and cancer, prior CABG and PCI, diabetes, dyslipidemia, hypertension, and hospital-level characteristics, including bed size, accreditation by The Joint Commission, adult cardiac surgery, adult cardiology services, adult diagnostic catheterization, adult interventional cardiac catheterization, rural referral center, sole community provider, teaching status, and interaction terms between sex and race/ethnicity to determine whether sex disparities vary across racial/ethnic groups. Model 3 also includes prior use of ezetimibe and PCSK9i.  * p-value for the interaction between sex and racial/ethnic groups among patients using a statin: 0.10. p-value for the interaction between sex and racial/ethnic groups among patients not using a statin: 0.34. | | | | |  |

**Supplemental Table 5.** Prevalence ratios for LDL-C <55 mg/dL at arrival associated with statin use, sex, and race/ethnicity, among those with LDL-C documented (n=23,558). Sensitivity analysis.

|  |  | Prevalence ratio (95% CI) | | | |
| --- | --- | --- | --- | --- | --- |
|  | LDL-C <55 mg/dL, n (%) | Model 1 | Model 2 | Model 3* | |
| Overall | 5,992 (25.4) | --- | --- | --- | |
| **By statin use** |  |  |  |  | |
| Using a statin | 4,945 (30.4) | 2.11 (1.97-2.25) | 2.07 (1.93-2.22) | 2.12 (1.96-2.29) | |
| Not using a statin | 1,047 (14.4) | 1 (ref) | 1 (ref) | 1 (ref) | |
| **By sex** |  |  |  |  | |
| **Using a statin** |  |  |  |  | |
| Men | 3.663 (31.2) | 1 (ref) | 1 (ref) | 1 (ref) | |
| Women | 1,281 (28.1) | 0.90 (0.85-0.95) | 0.89 (0.84-0.94) | 0.83 (0.77-0.89) | |
| **Not using a statin** |  |  |  |  | |
| Men | 785 (15.1) | 1 (ref) | 1 (ref) | 1 (ref) | |
| Women | 261 (12.7) | 0.84 (0.73-0.96) | 0.80 (0.70-0.92) | 0.75 (0.64-0.89) | |
| **By race/ethnicity** |  |  |  |  | |
| **Using a statin** |  |  |  |  | |
| Non-Hispanic white | 3,709 (32.8) | 1 (ref) | 1 (ref) | 1 (ref) | |
| Non-Hispanic Black | 452 (19.5) | 0.59 (0.54-0.65) | 0.66 (0.60-0.73) | 0.65 (0.57-0.74) | |
| Hispanic | 423 (28.2) | 0.86 (0.79-0.94) | 0.93 (0.85-1.01) | 0.90 (0.79-1.03) | |
| Other | 228 (32.2) | 0.98 (0.87-1.10) | 1.01 (0.91-1.13) | 0.91 (0.79-1.05) | |
| **Not using a statin** |  |  |  |  | |
| Non-Hispanic white | 797 (15.5) | 1 (ref) | 1 (ref) | 1 (ref) | |
| Non-Hispanic Black | 85 (9.7) | 0.62 (0.50-0.77) | 0.73 (0.58-0.91) | 0.70 (0.51-0.96) | |
| Hispanic | 79 (12.1) | 0.78 (0.62-0.98) | 0.83 (0.66-1.04) | 0.83 (0.62-1.10) | |
| Other | 38 (12.6) | 0.81 (0.61-1.09) | 0.87 (0.64-1.07) | 0.82 (0.56-1.20) | |
| BMI: body mass index; CABG: coronary artery bypass grafting; CI: confidence interval; LDL-C: low-density lipoprotein cholesterol; PAD: peripheral artery disease; PCI: percutaneous coronary intervention; PCSK9i: Proprotein Convertase Subtilisin/Kexin Type 9 inhibitor.  Model 1 is unadjusted.  Model 2 includes sex and race/ethnicity, simultaneously, with adjustment for calendar year, age, and geographic region.  Model 3 includes sex and race/ethnicity, with adjustment for calendar year, age, geographic region, payment source, unmet social needs, BMI, history of smoking, stroke, PAD, heart failure, and cancer, prior CABG and PCI, diabetes, dyslipidemia, hypertension, and hospital-level characteristics, including bed size, accreditation by The Joint Commission, adult cardiac surgery, adult cardiology services, adult diagnostic catheterization, adult interventional cardiac catheterization, rural referral center, sole community provider, teaching status, and interaction terms between sex and race/ethnicity to determine whether sex disparities vary across racial/ethnic groups. Model 3 also includes prior use of ezetimibe and PCSK9i.  * p-value for the interaction between sex and racial/ethnic groups among patients using a statin: 0.11. p-value for the interaction between sex and racial/ethnic groups among patients not using a statin: 0.17. | | | | |  |

**Supplemental Table 6**. Patient characteristics associated with LDL-C <70 mg/dL at admission, among those with LDL-C documented (n=23,558). Exploratory analysis.

|  |  | Prevalence ratio (95% CI) | |
| --- | --- | --- | --- |
|  | LDL-C <70 mg/dL  n (%) | Unadjusted | Fully adjusted |
| Year, n (%) |  |  |  |
| 2023 | 4,775 (41.9) | 1 (ref) | 1 (ref) |
| 2024 | 5,253 (43.2) | 1.03 (1.00-1.07) | 1.01 (0.98-1.05) |
| Age |  |  |  |
| 18 to 44 years | 142 (20.6) | 0.71 (0.61-0.83) | 0.76 (0.64-0.90) |
| 45 to 59 years | 1,435 (28.9) | 1 (ref) | 1 (ref) |
| 60 to 69 years | 2,737 (39.8) | 1.38 (1.31-1.45) | 1.25 (1.18-1.32) |
| 70 to 79 years | 3,465 (50.7) | 1.76 (1.68-1.84) | 1.47 (1.38-1.56) |
| ≥80 years | 2,249 (53.7) | 1.86 (1.77-1.96) | 1.55 (1.45-1.65) |
| Geographic region |  |  |  |
| Eastern | 2,489 (42.7) | 1 (ref) | 1 (ref) |
| Midwest | 1,638 (44.7) | 1.05 (0.97-1.13) | 1.00 (0.92-1.08) |
| Western | 1,632 (45.2) | 1.06 (0.99-1.14) | 0.97 (0.89-1.06) |
| Southeast | 1,879 (40.5) | 0.95 (0.88-1.03) | 1.00 (0.93-1.07) |
| Southwest | 2,387 (41.2) | 0.96 (0.91-1.03) | 0.92 (0.86-1.00) |
| Payment source |  |  |  |
| Traditional Medicare | 1,380 (45.9) | 1 (ref) | 1 (ref) |
| Medicare Advantage | 2,312 (48.3) | 1.05 (0.99-1.11) | 1.03 (0.97-1.10) |
| Other government insurance | 2,015 (40.2) | 0.88 (0.82-0.93) | 0.97 (0.91-1.04) |
| Private | 1,814 (35.9) | 0.78 (0.74-0.83) | 0.99 (0.93-1.06) |
| Government and private | 1,959 (50.7) | 1.10 (1.04-1.17) | 1.05 (0.99-1.11) |
| Uninsured | 280 (23.6) | 0.51 (0.45-0.58) | 0.76 (0.67-0.85) |
| Not documented | 48 (26.2) | 0.57 (0.44-0.74) | 0.74 (0.55-0.98) |
| Unmet social needs |  |  |  |
| Yes | 560 (33.2) | 0.75 (0.69-0.81) | 0.91 (0.84-0.99) |
| No | 5,888 (44.3) | 1 (ref) | 1 (ref) |
| Not assessed | 3,580 (41.7) | 0.94 (0.90-0.98) | 0.99 (0.95-1.03) |
| Body mass index |  |  |  |
| <18.5 Kg/m^2^ | 93 (42.1) | 0.96 (0.82-1.11) | 1.09 (0.93-1.27) |
| 18.5 to <25 Kg/m^2^ | 2,286 (44.0) | 1 (ref) | 1 (ref) |
| 25 to < 30 Kg/m^2^ | 3,622 (43.5) | 0.99 (0.95-1.03) | 0.97 (0.93-1.02) |
| ≥ 30 Kg/m^2^ | 3,836 (40.9) | 0.93 (0.89-0.97) | 0.96 (0.92-1.01) |
| History of smoking | 2,724 (34.6) | 0.74 (0.71-0.78) | 0.89 (0.84-0.93) |
| No history of smoking | 7,283 (46.5) | 1 (ref) | 1 (ref) |
| History of stroke | 1,185 (44.7) | 1.06 (1.00-1.12) | 1.00 (0.94-1.05) |
| No history of stroke | 8,843 (42.3) | 1 (ref) | 1 (ref) |
| History of PAD | 1,126 (47.7) | 1.14 (1.08-1.19) | 1.01 (0.97-1.06) |
| No history of PAD | 8,902 (42.0) | 1 (ref) | 1 (ref) |
| History of heart failure | 2,653 (46.4) | 1.12 (1.09-1.16) | 1.02 (0.98-1.06) |
| No history of heart failure | 7,375 (41.3) | 1 (ref) | 1 (ref) |
| History of cancer | 1,321 (52.1) | 1.26 (1.20-1.32) | 1.11 (1.06-1.16) |
| No history of cancer | 8,707 (41.4) | 1 (ref) | 1 (ref) |
| Prior CABG | 3,016 (48.5) | 1.20 (1.16-1.24) | 0.98 (0.94-1.01) |
| No prior CABG | 7,012 (40.4) | 1 (ref) | 1 (ref) |
| Prior PCI | 7,490 (42.9) | 1.03 (0.99-1.07) | 1.00 (0.96-1.04) |
| No prior PCI | 2,538 (41.7) | 1 (ref) | 1 (ref) |
| Diabetes | 5,130 (47.1) | 1.22 (1.18-1.25) | 1.16 (1.12-1.19) |
| Without diabetes | 4,898 (38.7) | 1 (ref) | 1 (ref) |
| Dyslipidemia | 8,293 (44.0) | 1.20 (1.15-1.25) | 0.94 (0.90-0.99) |
| Without dyslipidemia | 1,735 (36.7) | 1 (ref) | 1 (ref) |
| Hypertension | 9,004 (43.7) | 1.26 (1.19-1.33) | 1.06 (1.00-1.12) |
| Without hypertension | 1,024 (34.7) | 1 (ref) | 1 (ref) |
| Using ezetimibe |  |  |  |
| Yes | 957 (51.1) | 1.22 (1.16-1.28) | 1.13 (1.08-1.19) |
| No | 9,071 (41.8) | 1 (ref) | 1 (ref) |
| Using PCSK9i |  |  |  |
| Yes | 273 (57.1) | 1.35 (1.25-1.46) | 1.61 (1.46-1.78) |
| No | 9,755 (42.3) | 1 (ref) | 1 (ref) |
| **Hospital-level characteristics** |  |  |  |
| Bed size |  |  |  |
| Less than 100 beds | 345 (40.9) | 0.99 (0.89-1.11) | 1.03 (0.91-1.16) |
| 100-199 beds | 2,147 (45.5) | 1.10 (1.02-1.19) | 1.15 (1.08-1.23) |
| 200-299 beds | 2,238 (41.3) | 1 (ref) | 1 (ref) |
| 300-399 beds | 1,338 (43.9) | 1.06 (0.98-1.15) | 1.09 (1.01-1.18) |
| 400-499 beds | 910 (42.8) | 1.03 (0.94-1.14) | 1.12 (1.04-1.21) |
| 500 or more beds | 2,781 (41.1) | 0.99 (0.92-1.08) | 1.06 (0.98-1.14) |
| Accreditation by The Joint Commission |  |  |  |
| Yes | 8,287 (42.4) | 0.98 (0.90-1.06) | 1.03 (0.98-1.08) |
| No | 1,472 (43.4) | 1 (ref) | 1 (ref) |
| Adult cardiac surgery |  |  |  |
| Yes | 6,649 (42.6) | 1.03 (0.97-1.10) | 1.06 (0.99-1.14) |
| No | 1,932 (41.4) | 1 (ref) | 1 (ref) |
| Adult cardiology services |  |  |  |
| Yes | 8,458 (42.2) | 0.94 (0.81-1.10) | 0.93 (0.80-1.07) |
| No | 123 (44.7) | 1 (ref) | 1 (ref) |
| Adult diagnostic catheterization |  |  |  |
| Yes | 8,418 (42.2) | 0.94 (0.86-1.03) | 0.85 (0.68-1.05) |
| No | 163 (44.9) | 1 (ref) | 1 (ref) |
| Adult interventional cardiac catheterization |  |  |  |
| Yes | 8,427 (42.3) | 1.00 (0.88-1.15) | 1.15 (0.93-1.41) |
| No | 154 (42.1) | 1 (ref) | 1 (ref) |
| Rural referral center |  |  |  |
| Yes | 3,513 (43.5) | 1.03 (0.98-1.09) | 1.02 (0.98-1.07) |
| No | 6,246 (42.1) | 1 (ref) | 1 (ref) |
| Sole community provider |  |  |  |
| Yes | 214 (45.1) | 1.06 (0.93-1.21) | 1.09 (0.95-1.25) |
| No | 9,545 (42.5) | 1 (ref) | 1 (ref) |
| Teaching status* |  |  |  |
| Major | 1,732 (40.2) | 0.96 (0.89-1.03) | 0.94 (0.85-1.03) |
| Minor | 6,366 (43.4) | 1.03 (0.97-1.10) | 0.99 (0.94-1.05) |
| Non-teaching | 1,661 (42.0) | 1 (ref) | 1 (ref) |
| CABG: coronary artery bypass grafting; LDL-C: low-density lipoprotein cholesterol; PAD: peripheral artery disease; PCI: percutaneous coronary intervention; PCSK9i: Proprotein Convertase Subtilisin/Kexin Type 9 inhibitor; ref: reference; SD: standard deviation.  The unadjusted model includes each variable, one at a time.  The fully adjusted model includes all variables in the table, simultaneously, and statin use before admission, sex, race/ethnicity, and an interaction between sex and race/ethnicity.  * Teaching status: Major Teaching Hospitals - those with Council of Teaching Hospitals designation (COTH); Minor Teaching Hospitals - those approved to participate in residency and/or internship training by the Accreditation Council for Graduate Medical Education (ACGME), or the American Osteopathic Association (AOA); or those with medical school affiliation reported to the American Medical Association (AMA); Non-Teaching Hospitals - those without COTH, ACGME, AOA or Medical School (AMA) affiliation. | | | |

**Supplemental Table 7.** Lipid-lowering therapy regimens at admission among patients with LDL-C <70 mg/dL and ≥70 mg/dL.

| **Lipid-lowering therapy regimen** | **LDL-C <70 mg/dL**  n=10,028 | **LDL-C ≥70 mg/dL**  n=13,530 |
| --- | --- | --- |
| No lipid-lowering therapy, n (%) | 1,591 (15.9) | 5,006 (37.0) |
| Lipid-lowering regimen, n (%)* |  |  |
| Statin only | 7,283 (86.3) | 7,440 (87.3) |
| Ezetimibe only | 86 (1.0) | 276 (3.2) |
| PCSK9i only | 124 (1.5) | 115 (1.3) |
| Statin + ezetimibe | 795 (9.4) | 603 (7.1) |
| Statin + PCSK9i | 73 (0.9) | 53 (0.6) |
| Ezetimibe + PCSK9i | 47 (0.6) | 19 (0.2) |
| Statin + ezetimibe + PCSK9i | 29 (0.3) | 18 (0.2) |
| LDL-C: low-density lipoprotein cholesterol; PCSK9i: Proprotein Convertase Subtilisin/Kexin Type 9 inhibitor.  * Percentages for treatment combinations were calculated among patients receiving lipid-lowering therapy. | | |
